# Insult-dependent effect of bone marrow cell therapy on inflammatory response in a murine model of extrapulmonary acute respiratory distress syndrome

**DOI:** 10.1186/scrt334

**Published:** 2013-10-13

**Authors:** Tatiana Maron-Gutierrez, Johnatas Dutra Silva, Fernanda Ferreira Cruz, Samantha Alegria, Debora Gonçalves Xisto, Edson Fernandes Assis, Hugo Caire Castro-Faria-Neto, Claudia Chimisso Dos Santos, Marcelo Marcos Morales, Patricia Rieken Macedo Rocco

**Affiliations:** 1 Laboratory of Pulmonary Investigation, Carlos Chagas Filho Institute of Biophysics, Federal University of Rio de Janeiro, Avenida Carlos Chagas Filho, 373, Sala G1-014, Cidade Universitária, Rio de Janeiro, RJ, CEP:21.941-902, Brazil; 2 Laboratory of Cellular and Molecular Physiology, Carlos Chagas Filho Institute of Biophysics, Federal University of Rio de Janeiro, Rio de Janeiro, RJ, Brazil; 3 Laboratory of Immunopharmacology, Oswaldo Cruz Institute, FIOCRUZ, Rio de Janeiro, Avenida Brasil 4365, Pavilhão Ozório de Almeida, sala 16, RJ, CEP. 21040-900, Brazil; 4 The Keenan Research Centre of the Li Ka Shing Knowledge Institute of St. Michael's Hospital, 30 Bond Street, M5B 1W8, Toronto, Ontario, Canada; 5 Interdepartmental Division of Critical Care, University of Toronto, St. Michael’s Hospital, 30 Bond St., Toronto, Ontario, M5B 1W8, Canada

## Abstract

**Introduction:**

Administration of bone marrow-derived cells produces beneficial effects in experimental extrapulmonary acute respiratory distress syndrome (ARDS). However, there are controversies regarding the effects of timing of cell administration and initial insult severity on inflammatory response. We evaluated the effects of bone marrow-derived mononuclear cells (BMDMC) in two models of extrapulmonary ARDS once lung morphofunctional changes had already been installed.

**Methods:**

BALB/c mice received lipopolysaccharide (LPS) intraperitoneally (5 mg/kg in 0.5 ml saline) or underwent cecal ligation and puncture (CLP). Control mice received saline intraperitoneally (0.5 ml) or underwent sham surgery. At 24 hours, groups were further randomized to receive saline or BMDMC (2 × 10^6^) intravenously. Lung mechanics, histology, and humoral and cellular parameters of lung inflammation and remodeling were analyzed 1, 3 and 7 days after ARDS induction.

**Results:**

BMDMC therapy led to improved survival in the CLP group, reduced lung elastance, alveolar collapse, tissue and bronchoalveolar lavage fluid cellularity, collagen fiber content, and interleukin-1β and increased chemokine (keratinocyte-derived chemokine and monocyte chemotactic protein-1) expression in lung tissue regardless of the experimental ARDS model. Intercellular adhesion molecule-1 and vascular cell adhesion molecule-1 expression in lung tissue increased after cell therapy depending on the insult (LPS or CLP).

**Conclusions:**

BMDMC therapy at day 1 successfully reduced lung inflammation and remodeling, thus contributing to improvement of lung mechanics in both extrapulmonary ARDS models. Nevertheless, the different inflammatory responses induced by LPS and CLP resulted in distinct effects of BMDMC therapy. These data may be useful in the clinical setting, as they suggest that the type of initial insult plays a key role in the outcome of treatment.

## Introduction

Acute respiratory distress syndrome (ARDS) is a multifaceted syndrome with a wide-ranging clinical phenotype, making it a challenge to translate experimental results into feasible therapies in the clinical setting [1]. Several studies have addressed the therapeutic benefits of bone marrow-derived cells, such as bone marrow-derived mononuclear cells (BMDMCs) and mesenchymal stromal cells, in murine models of extrapulmonary ARDS induced by intraperitoneal administration of *Escherichia coli* lipopolysaccharide (LPS) [2,3] and cecal ligation and puncture (CLP) [4-6]. Bone marrow-derived cells have been shown to mitigate systemic and pulmonary inflammation, as well as decrease lung edema and enhance bacterial clearance, resulting in lower mortality [2,4-7]. Nevertheless, the potential effects of the severity of ARDS on inflammatory response and the time course for lung damage, which may play a role in the outcome of treatments [8], have not been addressed in the literature. It may also be argued that these effects are mediated by the specific characteristics of different types of injury.

In the present study, the beneficial effects of cell therapy were tested in light of the hypothesis that BMDMCs may exert distinctive effects on lung inflammation and remodeling depending on the initial insult. To this end, murine models of LPS and CLP-induced pulmonary injury [8] were used, and lung mechanics, histology, and humoral and cellular parameters of lung inflammation and remodeling were analyzed at 1, 3 and 7 days after induction of ARDS.

## Methods

This study was approved by the Ethics Committee of the Carlos Chagas Filho Institute of Biophysics, Health Sciences Center, and Federal University of Brazil (CEUA-CCS, IBCCF 019). All animals received humane care in compliance with the Principles of Laboratory Animal Care formulated by the National Society for Medical Research and the Guide for the Care and Use of Laboratory Animals prepared by the US National Academy of Sciences.

### Cell isolation

BMDMCs were isolated from the femurs and tibiae of 8-week-old BALB/c mice as described previously [2]. Briefly, bone marrow cells from male BALB/c mice were flushed from femurs and tibias with Dulbecco’s modified Eagle’s medium. After a homogeneous cell suspension was achieved, cells were centrifuged (400×*g* for 10 minutes), resuspended in Dulbecco’s modified Eagle’s medium and added to Ficoll-Hypaque. The isolated cells were counted in a Neubauer chamber with Trypan Blue for evaluation of viability. Saline or BMDMCs were injected into the jugular vein.

### Experimental protocol

**Figure 1:**
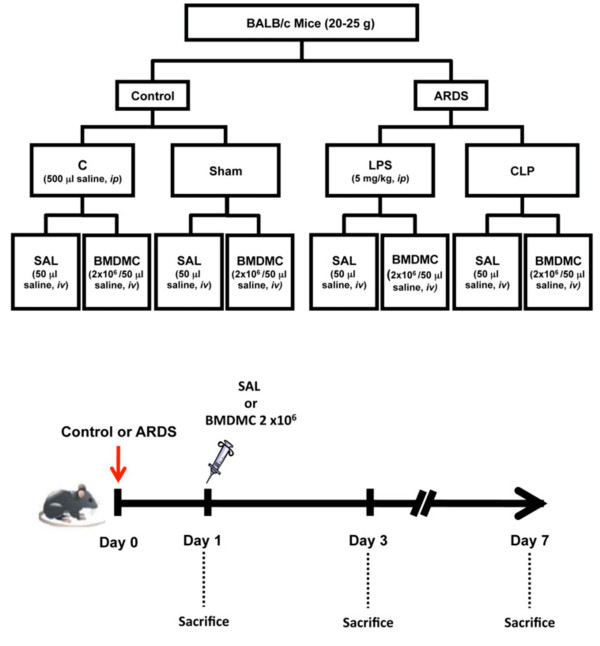
**Flowchart and timeline of study design.** Control (group C) animals received saline intraperitoneally (ip). LPS group animals received *Escherichia coli* lipopolysaccharide intraperitoneally. CLP group animals were subjected to cecal ligation and puncture. A sham*-*operated group was used as control for animals undergoing CLP. At day 1, some animals were sacrificed, post acute respiratory distress syndrome (ARDS), to evaluate lung function and histology, while other animals were further randomized into subgroups to receive saline (SAL) or bone marrow-derived mononuclear cells (BMDMCs, 2 × 10^6^ cells) intravenously (iv). Animals were euthanized at days 1, 3 and 7.

BALB/c mice (age 8 to 10 weeks) were used: 98 females and 10 male donors (weight 20 to 25 g). Numbers were given to each cage and letters were given to each experimental group for the randomization process, and afterwards each number was linked to a letter. A member of the laboratory that was blind to the experimental procedure performed the randomization process. Animals in the control groups received saline intraperitoneally (0.5 ml, group C) or were subjected to sham surgery (sham group). Mice in the ARDS groups received *E. coli* LPS intraperitoneally (5 mg/kg in 0.5 ml saline; LPS group) or underwent CLP (Figure 1). In the CLP groups, polymicrobial sepsis was induced as described previously [8]. Briefly, animals were anesthetized with sevoflurane and a midline laparotomy was performed. The cecum was carefully isolated and a 3–0 cotton ligature was placed below the ileocecal valve to prevent bowel obstruction. Finally, the cecum was punctured twice with an 18-gauge needle [8]. In the sham group, an abdominal incision was made with no cecal ligation and perforation. Both layers of the abdominal cavity were closed with 3.0 silk sutures, followed by fluid resuscitation with 0.5 ml/10 g body weight of prewarmed sterile saline subcutaneously. Sham and CLP animals received tramadol (0.05 mg/kg body weight subcutaneously) for postoperative analgesia, repeated every 8 hours. After this step, animals returned to their cages, where they received water and food *ad libitum*. All manipulations described were performed by the same investigator to ensure consistency. Twenty-four hours after lung injury, animals in group C and the ARDS group were further randomized to receive saline (0.05 ml) or BMDMCs (2 × 10^6^ BMDMCs in 0.05 ml saline) intravenously. At day 1, these models had similar degrees of lung mechanical compromise [8].

### Lung mechanics

On days 1, 3 and 7 after induction of ARDS, mice were sedated (diazepam 1 mg intraperitoneally), anesthetized (thiopental sodium 20 mg/kg intraperitoneally), tracheotomized, paralyzed (vecuronium bromide, 0.005 mg/kg intravenously), and mechanically ventilated with the following settings: respiratory frequency 100 breaths/minute, tidal volume 0.2 ml, and fraction of inspired oxygen 0.21. The anterior chest wall was surgically removed and a positive end-expiratory pressure of 2 cmH_2_O was applied. After a 10-minute ventilation period, lung mechanics were computed. At the end of the experiment (approximately 30 minutes), the lungs were prepared for histology and molecular biology. Airflow, volume and tracheal pressure were measured [9]. In an open chest preparation, tracheal pressure reflects transpulmonary pressure. Static lung elastance (Est,L) was computed using the ANADAT data analysis software (RHT-InfoData, Inc., Montreal, Quebec, Canada).

### Lung histology

Laparotomy was performed immediately after determination of lung mechanics. Heparin was injected intravenously, the trachea was clamped at end expiration, and the abdominal aorta and vena cava were sectioned. The right lung was removed, fixed in 4% buffered formaldehyde, embedded in paraffin, and cut into slices 4 μm thick, which were stained with hematoxylin and eosin (Vetec Química Fina, Rio de Janeiro, Brazil).

The volume fraction of collapsed and normal pulmonary areas and the number of macrophages and neutrophils in pulmonary tissue were determined by the point-counting technique at a magnification of ×200 across 10 random, noncoincident microscopic fields [10]. Collagen fibers (picrosirius polarization method) [11] were quantified in the alveolar septa and expressed as the percentage of collagen fibers per tissue area.

### Inflammatory mediators and growth factor mRNA expression

Quantitative real-time reverse transcription polymerase chain reaction was performed to measure the relative levels of mRNA expression of interleukin (IL)-1β, IL-6, IL-10, keratinocyte-derived chemokine (KC), monocyte chemotactic protein (MCP)-1, intercellular adhesion molecule (ICAM)-1, vascular cell adhesion molecule (VCAM)-1, and transforming growth factor beta (TGF-β). Slices were obtained from the center of the left lung, collected in cryotubes, quick-frozen by immersion in liquid nitrogen and stored at −80°C. Total RNA was extracted from the frozen tissues using the SV Total RNA Isolation System (Promega, Rio de Janeiro, Brazil) according to the manufacturer’s instructions. RNA concentrations were measured in a Nanodrop-® ND-1000 spectrophotometer (NanoDrop Technologies, Inc. 3411 Silverside Road. Bancroft Building. Wilmington, DE 19810 USA). First-strand cDNA was synthesized from total RNA using the GoTaq® 2-Step RT-qPCR System (Promega), according to the manufacturer’s recommendations. Relative mRNA levels were measured with a SYBR green detection system using a Mastercycler ep realplex^2^ S (Eppendorf, São Paulo, Brazil). All samples were measured in triplicate. The relative amount of expression of each gene was calculated as the ratio of studied gene to a control gene (acidic ribosomal phosphoprotein P0 (36B4)) and expressed as the fold-change relative to their respective control (group C or sham group).

The following polymerase chain reaction primers were used: IL-1β, forward 5′-GTT GAC GGA CCC CAA AAG-3′ and reverse 5′-GTG CTG CTG CGA GAT TTG-3′, 93 base pairs (bp); IL-6, forward 5′-TCT CTG GGA AAT CGT GGA A-3′ and reverse 5′-TCT GCA AGT GCA TCA TCG T-3′, 81 bp; IL-10, forward 5′-TCC CTG GGT GAG AAG CTG-3′ and reverse 5′-GCT CCA CTG CCT TGC TCT-3′, 91 bp; KC, forward 5′-TGA AGC TCC CTT GGT TCA G-3′ and reverse 5′-GGT GCC ATC AGA GCA GTC T-3′, 91 bp; MCP-1, forward 5′-CTT CTG GGC CTG CTG TTC A-3′ and reverse 5′-CCA GCC TAC TCA TTG GGA TCA-3′, 127 bp; ICAM-1, forward 5′-CCG CAG GTC CAA TTC ACA CT-3′ and reverse 5′-TCC AGC CGA GGA CCA TAC AG-3′, 143 bp; VCAM-1, forward 5′-GTG AAG ATG GTC GCC GTC TT-3′ and reverse 5′-GGC CAT GGA GTC ACC GAT T-3′, 126 bp; TGF-β, forward 5′-ATA CGC CTG agt GGC TGT C-3′ and reverse 5′-GCC CTG TAT TCC GTC TCC T-3′, 77 bp; and 36B4 (Rplp0), forward 5′-CAA CCC AGC TCT GGA GAA AC-3′ and reverse 5′-GTT CTG AGC TGG CAC AGT GA-3′, 150 bp.

### Inflammatory mediators and growth factor protein expression

IL-1β, IL-6, IL-10, KC, MCP-1, and TGF-β protein expressions were measured with ELISA kits accordingly to the manufacturer's instructions (R&D Systems, Minneapolis, MN, USA).

### Bronchoalveolar lavage fluid

Bronchoalveolar lavage was carried out in the left lung via a tracheal tube with phosphate-buffered saline solution (0.5 ml) containing ethylenediamine tretraacetic acid (10 mM). Total leukocyte numbers were measured in a Neubauer chamber under light microscopy after diluting the samples in Türk solution (2% acetic acid). Bronchoalveolar lavage fluid was centrifuged at 4°C for 10 minutes at 400×*g* and the cell pellet resuspended in phosphate-buffered saline for further leukocyte enumeration. The protein concentration was determined by the Bradford method. Differential cell counts were performed in cytospin smears stained by the May–Grünwald–Giemsa method [2,3].

### Statistical analysis

Comparison between control and sham groups was performed at all endpoints (days 1, 3 and 7) using two-way analysis of variance. Since lung function and histological data were not significantly different at these time points, we decided to show only one control group. Between-group differences were therefore assessed using one-way analysis of variance followed by Bonferroni’s *post-hoc* test. Survival curves were derived by the Kaplan–Meier method and compared by log-rank test. The significance level was set at 5%. All tests were performed in GraphPad Prism 5.0 (GraphPad Software, San Diego, CA, USA).

## Results

### Survival curve

**Figure 2:**
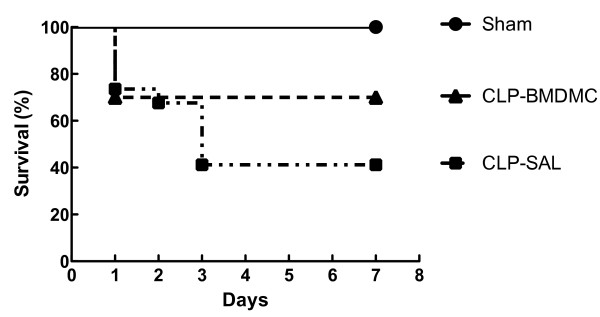
**Survival curves.** Survival curves in animals after bone marrow-derived mononuclear cells (BMDMC) or saline (SAL) treatment after induction of sepsis by cecal ligation and puncture (CLP) and in their respective control (sham) over 7 days. In the CLP-BMDMC group, the survival rate was 70% on day 7.

At day 7, the survival rate of groups C, LPS-SAL and LPS-BMDMC was 100%. Similarly, the sham mice survival rate was 100%. The CLP-SAL group had a survival rate of 40%. BMDMC administration improved survival in CLP animals (70% at day 7) (*P* <0.05) (Figure 2). Even though the mortality rate in the LPS-treated and CLP-treated animals was not comparable, the degrees of lung mechanical compromise were similar.

### BMDMC administration improved lung mechanics and morphometry in both models of extrapulmonary ARDS

**Figure 3:**
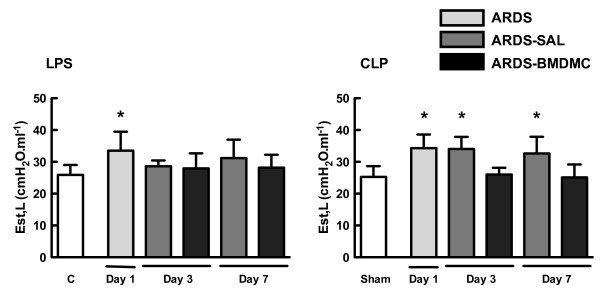
**Static lung elastance on days 1, 3 and 7.** Mice received *Escherichia coli* lipopolysaccharide (LPS) intraperitoneally or were subjected to cecal ligation and puncture (CLP). The control group for LPS received saline intraperitoneally, whereas a sham*-*operated group was used as control for animals undergoing CLP. At day 1, some animals were sacrificed, post acute respiratory distress syndrome (ARDS), in order to evaluate the static lung elastance (Est,L), while other animals were randomized and received saline (SAL) or bone marrow-derived mononuclear cells (BMDMC, 2×10^6^ cells) intravenously. Values expressed as mean ± standard deviation of six animals in each group. *Significantly different from the corresponding control group (C or sham) (*P* <0.05). ^#^ARDS-BMDMC vs*.* ARDS-SAL (*P* <0.05).

Est,L was higher in LPS animals than in controls on day 1. Nevertheless, Est,L in LPS-SAL mice was not increased compared with controls at days 3 and 7 (Figure 3). Est,L was higher in CLP animals than in sham animals on day 1. Est,L remained elevated in CLP-SAL mice at days 3 and 7. BMDMC administration led to a significant reduction in Est,L at days 3 and 7 (Figure 3).

**Figure 4:**
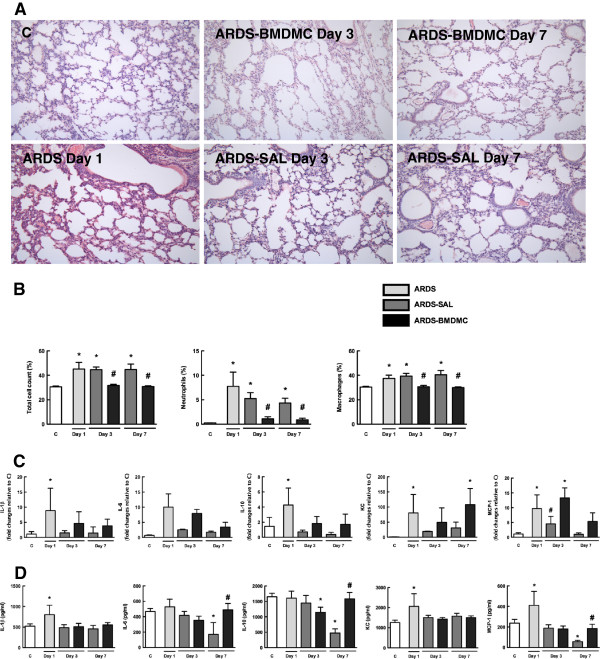
**Lung injury and inflammation in lipopolysaccharide-induced acute respiratory distress syndrome.** Representative photomicrographs (×200 magnification) of lung tissue stained with hematoxylin and eosin **(A)**, total and differential cell count in alveolar septa **(B)**, and levels of interleukin (IL)-1β, IL-6 and IL-10 and chemokines keratinocyte-derived chemokine (KC or CXCL1) and monocyte chemotactic protein-1 (MCP-1 or CCL2) mRNA **(C)** and protein expressions **(D)** in lung tissue. Values expressed as mean ± standard deviation of six animals **(B)** and of four to six animals **(C, D)** per group. *Significantly different from corresponding control group **(C)** (*P* <0.05). ^#^ARDS-BMDMC vs*.* ARDS-SAL (*P* <0.05). ARDS, acute respiratory distress syndrome; BMDMC, bone marrow-derived mononuclear cell; SAL, saline.

**Figure 5:**
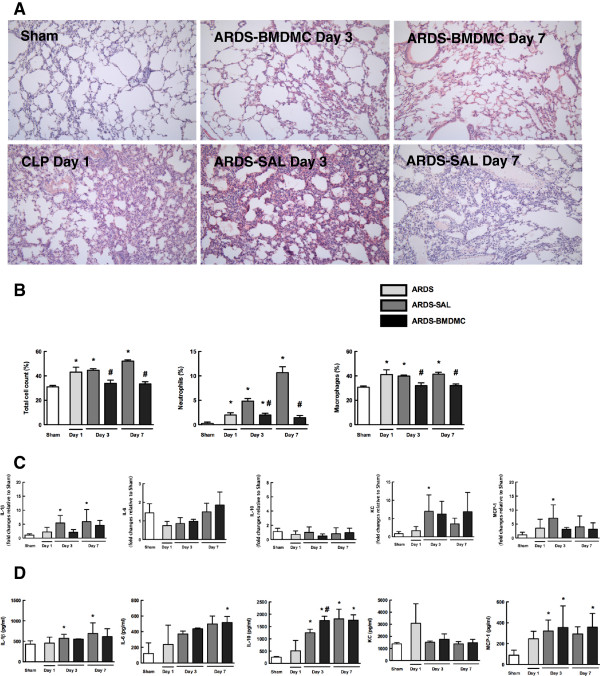
**Lung injury and inflammation in cecal ligation and puncture-induced acute respiratory distress syndrome.** Representative photomicrographs (×200 magnification) of lung tissue stained with hematoxylin and eosin **(A)**, total and differential cell count in alveolar septa **(B)**, and levels of interleukin (IL)-1β, IL-6 and IL-10 and chemokines keratinocyte-derived chemokine (KC or CXCL1) and monocyte chemotactic protein-1 (MCP-1 or CCL2) mRNA **(C)** and protein expressions **(D)** in lung tissue. Values expressed as mean ± standard deviation of six animals **(B)** and four to six animals **(C, D)** per group. *Significantly different from corresponding control group (sham) (*P* <0.05). ^#^ARDS-BMDMC vs*.* ARDS-SAL (*P* <0.05). ARDS, acute respiratory distress syndrome; BMDMC, bone marrow-derived mononuclear cell; SAL, saline.

At day 1, both LPS and CLP groups presented interstitial edema, formation of hyaline membrane, neutrophil infiltration and alveolar collapse (Figures 4 and 5). The fraction area of alveolar collapse was higher both in LPS (Figure 4A, Table 1) and in CLP (Figure 5A, Table 1) untreated mice at days 1, 3 and 7. BMDMC administration led to a reduction in alveolar collapse at days 3 and 7, regardless of the initial insult implicated in the pathogenesis of ARDS (Figures 4A and 5A, Table 1).

**Table 1:** Morphometric parameters

	**Group**	**Normal area (%)**		**Alveolar collapse (%)**		**Hyperinflation (%)**	
		**LPS**	**CLP**	**LPS**	**CLP**	**LPS**	**CLP**
	C (LPS) or sham (CLP)	99.6 ± 0.7	99.0 ± 1.2	0.4 ± 0.7	1.0 ± 1.2	0.0 ± 0.0	0.0 ± 0.0
Day 1	ARDS	85.0 ± 7.0*	92.0 ± 3.3*	13.4 ± 6.0*	5.6 ± 4.0*	1.6 ± 3.0	0.0 ± 0.0
Day 3	ARDS-SAL	93.5 ± 2.4*	86.1 ± 4.2*	6.5 ± 2.4*	11.0 ± 1.7*	0.0 ± 0.0	2.9 ± 4.2*
	ARDS-BMDMC	97.4 ± 1.5	94.6 ± 2.5#	2.6 ± 1.5	4.6 ± 2.4#	0.0 ± 0.0	0.0 ± 0.0
Day 7	ARDS-SAL	94.0 ± 1.0*	93.6 ± 2.9*	4.8 ± 2.0*	6.5 ± 2.8*	0.0 ± 0.0	0.3 ± 1.2
	ARDS-BMDMC	98.8 ± 0.8	96.8 ± 2.2	1.2 ± 0.8	3.2 ± 2.2	0.0 ± 0.0	0.0 ± 0.0

Mice received *Escherichia coli* lipopolysaccharide (LPS) intraperitoneally or were subjected to cecal ligation and puncture (CLP) surgery. Control group animals received saline intraperitoneally, whereas a sham*-*operated group was used as control for animals undergoing CLP. At day 1, some animals were sacrificed, post acute respiratory distress syndrome (ARDS), to evaluate lung morphometry, while other animals were further randomized into subgroups to receive saline (SAL) or bone marrow-derived mononuclear cells (BMDMC, 2×10^6^ cells) intravenously. Values expressed as mean ± standard deviation of sic animals in each group. All values were computed in 10 random, noncoincident fields per animal. *Significantly different from corresponding control group (control or sham) (*P* <0.05). ^#^ARDS-BMDMC vs*.* ARDS-SAL (*P* <0.05).

Neutrophil counts in lung tissue were increased in LPS mice compared with controls at all time points, most prominently on day 1 (Figure 4B). BMDMC administration reduced neutrophils at days 3 and 7 (Figure 4B). Macrophages and total cell counts in lung tissue were increased in LPS mice compared with controls at days 1, 3 and 7 (Figure 4B). Macrophages and total cell counts were decreased in LPS-BMDMC animals at days 3 and 7 (Figure 4B). In untreated CLP mice, the number of neutrophils in lung tissue was higher than in sham mice at all time points, most prominently on day 7 (Figure 5B). BMDMC administration reduced neutrophil counts at day 7 (Figure 5B). Macrophage and total cell counts in lung tissue were higher in CLP mice than in sham mice at days 1, 3 and 7 (Figure 5B). BMDMC administration reduced macrophage and total cell counts at days 3 and 7 (Figure 5B).

### Effects of BMDMCs on inflammatory mediators in the lung

The increase in inflammatory cell count in lung tissue was accompanied by a significant increase in lung tissue levels of inflammatory mediator mRNA expression in the saline-treated groups. IL-1β and IL-10 were increased on day 1 in the LPS-SAL group. Nevertheless, this increase was not sustained over time (Figure 4C). In CLP-SAL, the increase in IL-1β mRNA expression was observed at a later time point, at days 3 and 7 (Figure 5C). No significant differences were observed in IL-10 mRNA expression in the CLP-SAL group (Figure 5C) and in IL-6 mRNA expression in both CLP and LPS groups. BMDMCs reduced IL-1β mRNA expression in the LPS groups (Figure 4C) and CLP groups (Figure 5C). Levels of MCP-1 and KC mRNA expression were high on day 1 in the LPS-SAL group (Figure 4C), whereas in the CLP-SAL group both KC and MCP-1 were increased on day 3 (Figure 5C). In LPS-BMDMC mice, cell therapy augmented MCP-1 mRNA expression on day 3 and KC mRNA expression on day 7 (Figure 4C).

We also analyzed protein expressions of IL-1β, IL-6, IL-10, KC and MCP-1. At day 1 there was an increase in IL-1β, KC and MCP-1 protein expression, while at day 7 there was a decrease in IL-6, IL-10, KC and MCP-1 protein expressions in the LPS-SAL group (Figure 4D). Interestingly, at day 7 BMDMC therapy led to an increase of these mediators’ levels compared with LPS-SAL, returning to control values (Figure 4D). In the CLP-SAL group, there was an increase in IL-1β and IL-10 at days 3 and 7, and in MCP-1 at day 3 (Figure 5D). In CLP-BMDMC mice, cell therapy increased IL-6 expression at day 7, and IL-10 and MCP-1 at days 3 and 7.

### Effects of BMDMCs on influx of inflammatory cells and protein permeability in the alveolus

**Figure 6:**
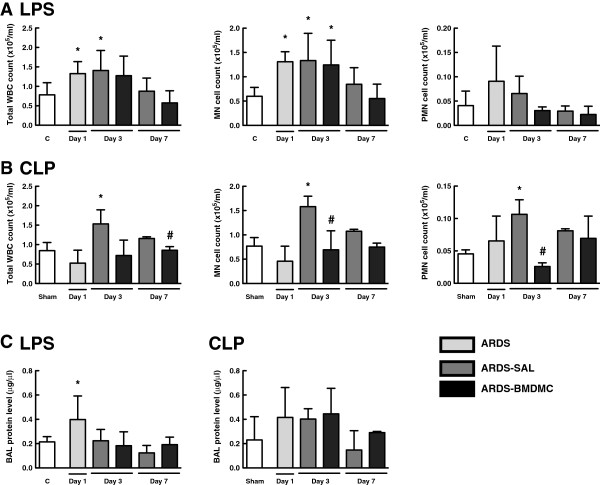
**Total and differential cell count and protein level in bronchoalveolar lavage fluid.** Total white blood cell (WBC) and differential cell count in bronchoalveolar lavage fluid (BALF) in the lipopolysaccharide (LPS) **(A)** and cecal ligation and puncture (CLP) **(B)** groups, as well as BALF protein levels **(C)**. Values expressed as mean ± standard deviation of four to six animals per group. *Significantly different from corresponding control group (C or sham) (*P* <0.05). ^#^ARDS-BMDMC vs*.* ARDS-SAL (*P* <0.05). ARDS, acute respiratory distress syndrome; BMDMC, bone marrow-derived mononuclear cell; MN, mononuclear; PMN, polymorphonuclear; SAL, saline.

The increase in lung tissue levels of inflammatory mediators was accompanied by a significant increase in inflammatory cell count in bronchoalveolar lavage fluid (BALF) (Figure 6A,B) and an increase in lung protein permeability (Figure 6C). In LPS groups, the total number of cells in BALF was increased at days 1 and 3 (Figure 6A). BMDMC administration resulted in a decrease in total cell infiltration in BALF. Mononuclear cells were increased at days 1 and 3, without significant differences in polymorphonuclear cell count (Figure 6A). In CLP groups there was an increase in the cell influx at day 3, both mononuclear cells and polymorphonuclear cells (Figure 6B). BMDMC therapy reduced mononuclear and polymorphonuclear cell influx at day 3 (Figure 6B).

### Effects of BMDMCs on pulmonary adhesion molecule mRNA expression are dependent on the initial insult

**Figure 7:**
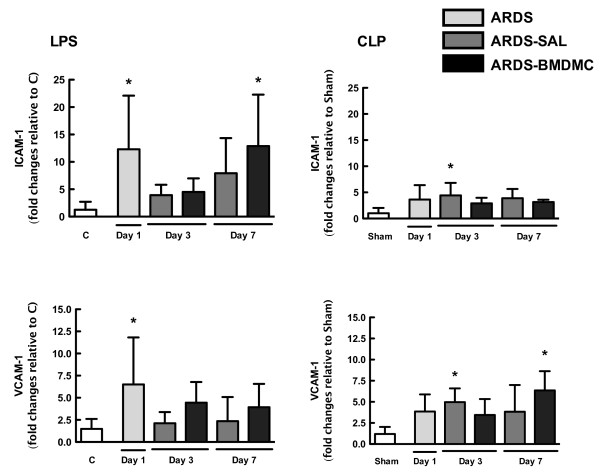
**Intracellular adhesion molecule-1 and vascular cell adhesion molecule-1.** Intracellular adhesion molecule (ICAM)-1 and vascular cell adhesion molecule (VCAM)-1 levels in lung tissue after lipopolysaccharide (LPS)-induced and cecal ligation and puncture (CLP)-induced acute respiratory distress syndrome (ARDS). Values expressed as mean ± standard deviation of four animals per group. *Significantly different from corresponding control group (C or sham) (*P* <0.05). BMDMC, bone marrow-derived mononuclear cell; SAL, saline.

**Figure 8:**
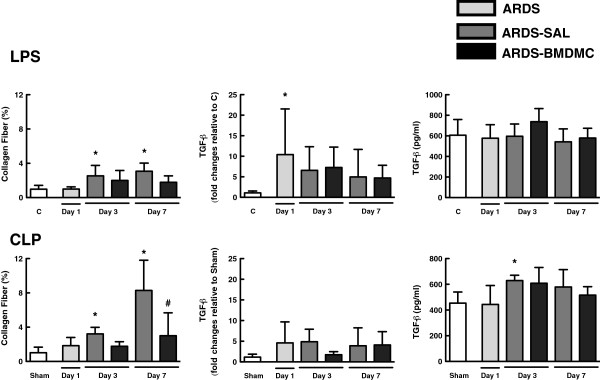
**Collagen fiber and transforming growth factor beta.** Collagen fiber content in alveolar septa and transforming growth factor beta (TGF-β) mRNA and protein levels in lung tissue after lipopolysaccharide (LPS)-induced and cecal ligation and puncture (CLP)-induced acute respiratory distress syndrome (ARDS). Values are mean ± standard deviation of four to six animals per group. *Significantly different from corresponding control group (C or sham) (*P* <0.05). ^#^ARDS-BMDMC vs*.* ARDS-SAL *(P <*0.05). BMDMC, bone marrow-derived mononuclear cell; SAL, saline.

Levels of ICAM-1 and VCAM-1 mRNA expression were high on day 1 in the LPS-SAL group (Figure 7), whereas in the CLP-SAL group these adhesion molecules were increased on day 3 (Figure 7). In LPS-BMDMC mice, cell therapy increased ICAM-1 mRNA expression on day 7 (Figure 7), with no significant differences in VCAM-1 mRNA expression; however, in CLP-BMDMC mice, VCAM-1 was increased on day 7 (Figure 8), without any increase in ICAM-1 mRNA expression.

### BMDMCs reduced collagen fiber content in the lung parenchyma

Both LPS-SAL and CLP-SAL mice had increased collagen fiber content at days 3 and 7 (Figure 8). BMDMC administration led to a significant decrease in collagen content; nevertheless, these changes in lung parenchyma were not associated with TGF-β mRNA and protein expression in lung tissue (Figure 8).

## Discussion

In the present study, in support of our initial hypothesis, BMDMC administration had distinct effects on adhesion molecule mRNA expression levels depending on the primary insult, resulting in an increase in ICAM-1 after LPS-induced ARDS, while increasing VCAM-1 levels after CLP-induced ARDS at day 7. This is the first study to compare cell therapy effects in two different experimental models of extrapulmonary ARDS (LPS and CLP). In previous studies from our group [2,3] we observed that cell therapy had distinct effects on lung inflammation and remodeling depending on ARDS etiology (pulmonary vs. extrapulmonary). In this context, we hypothesized that even in the same etiology of ARDS, depending on the primary insult (LPS and CLP), cell therapy could have beneficial yet distinct effects. Demonstrating this hypothesis, in the clinical scenario when patients are treated according to ARDS etiology (pulmonary vs. extrapulmonary), we should be aware of therapy effects, since there may be different outcomes related to lung inflammation and remodeling.

In previous studies with LPS-induced ARDS [2,7,12,13] and CLP-induced ARDS [4-6], bone marrow cells were administered only a few hours after injury. This administration limits the applicability of results to clinical practice, because the time course for lung damage is not taken into consideration. In order to produce a more clinically relevant scenario, in the present study BMDMCs were injected intravenously 1 day after the initial insult (LPS or CLP), once the lung mechanical and morphologic changes had already been established [6,8,14,15]. The effects of BMDMC therapy were evaluated at days 3 and 7. We are aware that most previous studies of ARDS have used mesenchymal stromal cells; nevertheless, mesenchymal stromal cells require culture conditions that are detrimental for cell transplantation and pose a risk of contamination and immunological reactions. Bearing these limitations in mind, and on the basis of some promising results from previous research conducted by our group [2,6,12,16,17], we employed BMDMCs because they can be easily and safely administered on the day of harvest and are known to reduce lung inflammation and fibrogenesis and thus restore lung function [2,6,12,17,18]. Because it has already been demonstrated that stem cell engraftment is not required for BMDMCs to exert their beneficial effects [2,4,6,16,17], we did not address this issue.

Several studies have suggested that the endothelium plays an important role in the pathophysiology of sepsis [19]. Our results suggest endothelial cell activation by increased expression of cell adhesion molecules and increased production of cytokines. Adhesion molecules (ICAM-1 and VCAM-1) of endothelial cells interact with integrins expressed on neutrophils that stabilize cell–cell interactions, providing firm adherence and thus facilitating cell transmigration [20]. ICAM-1 mRNA expression increased after intratracheal administration of endotoxin or CLP-induced ARDS, in accordance with previous studies [21-23]. Nevertheless, despite the increase in mRNA expression of ICAM-1 and VCAM-1 as well as chemokines (MCP-1 and KC), BMDMC administration resulted in fewer mononuclear and polymorphonuclear cells within the lung parenchyma. Nevertheless, we observed an increase in BALF mononuclear cells in both LPS-SAL and LPS-BMDMC groups at day 3. Additionally, when KC protein expression was analyzed we did not observe an increase after BMDMC therapy, in accordance with our polymorphonuclear cell count findings.

BMDMC administration reined in the inflammatory process, reducing inflammatory cell counts and modulating the levels of inflammatory mediators (such as IL-1β and IL-10) in lung tissue. IL-10 appears to play a pivotal role in controlling the magnitude of inflammatory responses during either systemic or pulmonary inflammation, and has been proposed as a key mediator of beneficial effects in CLP-induced experimental sepsis [5]. Nevertheless, other studies investigating bone marrow cell therapy, including those previously conducted by our group, suggest that the beneficial effects of BMDMC extend beyond an effect on a single mediator [2,4,6]. IL-1β is mainly produced by activated macrophages as a pro-peptide and is cleaved by caspase-1 within the inflammasome into an active enzyme [24]. IL-1β can upregulate cell-surface expression of leukocyte and vascular adhesion molecules and stimulate the production of chemokines required for leukocyte recruitment and activation [25]. Increased levels of IL-1β in ARDS patients correlate with development of pulmonary fibrosis [17,26]. In this context, we observed that BMDMC decreased lung collagen fiber content both in the LPS and CLP groups. Nevertheless, this reduction was not associated with differences in TGF-β mRNA and protein expressions in lung tissue, suggesting another signaling pathway (for example, collagenases) [3].

Studies using endotoxin models for the induction of sepsis have provided a wealth of important knowledge. Nevertheless, the clinical relevance of the LPS experimental model has been questioned, as administration of a bolus of endotoxins does not reflect the complex pathophysiology of sepsis [27]. Alternative experimental models of sepsis have thus been proposed [28], such as CLP, a model of polymicrobial sepsis [27,29,30]. Polymicrobial sepsis and endotoxemia have been reported to have differing temporal cytokine responses, innate defense mechanisms, and signaling molecules involved [31], which is consistent with our findings. Additionally, as recently defined by the American Thoracic Society [32], the diagnosis of ARDS in animals should be based on at least three of four main features of evidence: histology, inflammation, physiological dysfunction, and permeability. Our experimental models of LPS and CLP produced histological evidence of lung injury, inflammatory response and physiological dysfunction, meeting the American Thoracic Society criteria.

In this context, our study has some limitations. First, mortality was higher in the ARDS-CLP group (60%) than in the ARDS-LPS group (0%). Second, the CLP model is one in which there is an ongoing septic insult, with worsening injury over time. In contrast, the LPS model is one of a single insult, making it difficult to compare BMDMC effects across models. We plan future studies to compare the BMDMC therapy in two bacterial (septic) models. Third, inflammatory mediators were measured in lung tissue. The balance between local and systemic inflammation was therefore not evaluated. Fourth, collagen fiber content was evaluated using a histology technique, but future studies should be performed using either hydroxyproline or soluble collagen assay. Fifth, the study endpoint was 7 days after LPS administration or CLP for induction of ARDS. Nonetheless, to better evaluate the effects of BMDMC therapy on lung inflammation and remodeling, further studies of later time points would be interesting. Sixth, previous studies have used fibroblasts as control cells for cell therapy studies [7,13,33]; however, some have demonstrated that administration of fibroblasts, mesenchymal cells without stem cell properties, may not only be ineffective but may worsen injury [7,13,33,34]. Accordingly, saline was chosen as the control. Finally, the effects of BMDMCs on adhesion molecules might be of interest; however, further research, such as the use of knockout mice, is required to assess this issue.

## Conclusions

Specific beneficial effects of BMDMC therapy were observed for each type of initial insult triggering extrapulmonary ARDS. BMDMC therapy at day 1, once lung mechanical and histological changes had already been installed in ARDS, successfully reduced inflammation and remodeling in both extrapulmonary ARDS models. These data may be useful in the clinical setting, as they suggest that the type of insult plays an essential role in the outcome of ARDS treatment.

## Abbreviations

ARDS: Acute respiratory distress syndrome; BALF: Bronchoalveolar fluid lavage fluid; BMDMC: Bone marrow-derived mononuclear cell; bp: Base pairs; CLP: Cecal ligation and puncture; Est,L: Static lung elastance; ICAM-1: Intracellular adhesion molecule-1; IL: Interleukin; KC: Keratinocyte-derived chemokine; LPS: Lipopolysaccharide; MCP-1: Monocyte chemotactic protein-1; TGF-β: Transforming growth factor beta; VCAM-1: Vascular cell adhesion molecule-1.

## Competing interests

The authors declare that they have no competing interests.

## Authors’ contributions

TM-G made substantial contributions to the conception and design of the study, carried out the study, performed all animal experiments and collected all data, performed the statistical analysis, and drafted the manuscript. JDS measured inflammatory mediator, growth factor mRNA expression, BALF measurements, and drafted the manuscript. FFC and SA performed the histological analysis and drafted the manuscript. DGX made substantial contributions to the conception and design of the study, co-supervised the study, performed animal experiments and corrected the manuscript. EFA performed enzyme-linked immunosorbent assay experiments. HCC-F-N, CCDS, MMM and PRMR made substantial contributions to the conception and design and supervised the conduct of the study, and writing of the article. All authors read and approved the final manuscript.
